# Developmental Cascades from Aggression to Internalizing Problems via Peer and Teacher Relationships from Early to Middle Adolescence

**DOI:** 10.1007/s10964-021-01396-1

**Published:** 2021-02-02

**Authors:** Aja Louise Murray, Ingrid Obsuth, Lydia Speyer, George Murray, Karen McKenzie, Manuel Eisner, Denis Ribeaud

**Affiliations:** 1 Violence Research Centre, Institute of Criminology, University of Cambridge, Cambridge, UK; 2 Department of Psychology, University of Edinburgh, Edinburgh, UK; 3 Clinical Psychology Department, University of Edinburgh, Edinburgh, UK; 4 Department of Psychology, Northumbria University, Newcastle upon Tyne, UK; 5 Jacobs Center for Productive Youth Development, University of Zurich, Zurich, Switzerland

**Keywords:** Developmental cascade, Internalizing, Aggression, Teacher Relationships, Peer Relationships

## Abstract

Previous research has provided evidence for developmental cascades between externalizing and internalizing problems via mechanisms such as peer and academic problems; however, there remains a need to illuminate other key mediating processes that could serve as intervention targets. This study, thus, evaluated whether developmental associations between aggression and internalizing are mediated by teacher—as well as peer—relationships. Using data from z-proso, a longitudinal study of Swiss youth (*n* = 1523; 785 males), an autoregressive latent trajectory model with structured residuals (ALT-SR) was fit over ages 11, 13, and 15 to examine within-person developmental links between aggression, internalizing problems, and the mediating role of peer and teacher relationships, while disaggregating between- and within-person effects. Teacher and peer relationships did not play a role in the progression of externalizing to internalizing problems or vice versa, however, teacher and peer relationships showed a protective effect against developing internalizing problems at ages 13. The results suggest that good quality relationships with teachers in early adolescence can help prevent internalizing problems from developing.

## Introduction

There is substantial comorbidity between internalizing and externalizing problems across childhood and adolescence (Murray et al., 2016). Understanding the mechanisms of their relations within individuals over development is essential for identifying key intervention targets (Masten & Cicchetti, 2010) and adolescence is a period of heightened risk for the emergence or escalation of both externalizing (Barbot & Hunter, 2012) and internalizing problems (Rapee et al., 2019). This study, thus, evaluated the developmental associations between internalizing and an important form of externalizing, namely aggression, in adolescence and examined two candidate mediators of their links: teacher and peer relationships. An autoregressive latent trajectory model with structured residuals (ALT-SR) was employed to overcome a major limitation of the cross-lagged panel models traditionally used in this research area, namely, their conflation of within- and between-person effects.

In developmental approaches to comorbidity, the pathways linking externalizing and internalizing problems have been conceptualized in terms of developmental cascade models. These models suggest that links between domains of psychosocial functioning can emerge through problems in one domain having a causal effect on another (Masten & Cicchetti, 2010). Within this framework, the “dual failure model” proposed by Capaldi (1992) holds that externalizing problems lead to failures in the academic and social domains resulting in negative self-appraisals and low self-esteem and, in turn, to an increased risk of anxiety and depression. On the other hand, the “acting out model” proposed by Carlson and Cantwell (1980) holds that children and adolescents who experience anxiety and depression, may “act out” to express their distress; this may alienate them from prosocial friends and lead to conflict at home, which in turn escalates their externalizing behaviors. A large body of longitudinal research has found at least partial support for the dual failure model in both childhood and adolescence (e.g., Blain-Arcaro & Vaillancourt, 2017) and there is some evidence for cascades in the opposite direction, in line with the acting out model (e.g., Yu et al., 2018).

Peer problems such as rejection and victimization alongside academic problems have been the most commonly tested internalizing-externalizing developmental cascade mediators; however, they have not been found to fully account for the links between these two domains (e.g., van Lier et al., 2012). Indeed, there may be many further intermediaries that are responsible for the linkages between externalizing and internalizing problems. In the relationships domain, for example, peer relations may be especially significant during adolescence, owing to a heightened importance of peers in this period (Steinberg & Monahan, 2007); however, they are not the only relationships in adolescence that are likely to both be impacted by and impact internalizing problems and externalizing problems. Parental, teacher, and intimate partner relationships, in particular, are also likely to be important. Regarding parental relationships, one previous study examined the role of maternal dissatisfaction in mediating the developmental relations between externalizing and internalizing problems, alongside peer (bullying victimization), and academic problems (Wertz et al., 2015) and found it to be a significant mediator of a cascade from externalizing problems to internalizing problems. That study, however, only spanned the developmental period from age 5 to 12 and therefore only covered early adolescence. Further research is needed to examine the roles of significant relationships beyond peers in the longitudinal links between externalizing and internalizing problems across adolescence.

Based on transactional theory, teacher relationships are a strong candidate for an additional relationship-based mediator of internalizing-externalizing problem cascades in adolescence. Transactional theory holds that student socioemotional problems are reciprocally related to student-teacher relationships such that problems like externalizing behavior evoke negative reactions from teachers and undermine the formation of warm, supportive teacher-student relationships. According to attachment theory, warm and supportive bonds between youth and significant adults contributes to positive developmental outcomes and student-teacher relationships lacking these qualities have been proposed, by implication, to increase the risk of internalizing and externalizing problems (Pakarinen et al., 2018). Longitudinal evidence supports the contention that qualities of teacher relationships are reciprocally related to adolescent socioemotional problems. One recent study, for example, used a cross-lagged panel modeling approach to examine the developmental relations between teacher conflict and externalizing problems (Pakarinen et al., 2018). They found that externalizing problems in grade 4 were related to greater teacher conflict; however, there was no evidence for an effect of teacher conflict on the escalation of externalizing problems. Another recent study using a similar approach found that a good teacher relationship at age 15; characterized by a student feeling fairly treated by their teacher, trusting their teacher, and perceiving that their teacher makes sure there is no violence between students, was related to lower delinquency levels at age 16 (Theimann, 2016). However, there was no such effect across the age 13 to 14 or age 14 to 15 lags. The same study found a significant effect of a bad teacher relationship at age 13, characterized by a student feeling that teachers do not care about their problems and that the teacher tends to look away when it comes to severe fights between students, on delinquency at 14. There was no such effect at the age 14 to 15 or age 15 to 16 lag and no significant effect of delinquency on bad student-teacher relationships. Finally, a study using data from the current sample used a propensity score analysis approach to account for potential confounding and found that children who reported a more positive relationship with their teacher (characterized by getting along well with their teacher, feeling helped by their teacher, and feeling fairly treated by their teacher) compared to their matched pairs at age 10/11 showed less aggression at ages 11, 13, and 15 (Obsuth et al., 2017). Few studies have addressed the role of teacher relationships in internalizing problems in adolescence and evidence is somewhat mixed. One cross-lagged panel study found evidence consistent with the stress-buffer model, reporting that emotional support from teachers benefitted adolescents with average or high levels of stressful life events; however, those with low levels of stressful life events actually showed later increased depressive symptoms (Pössel et al., 2013). Another study of similar design; however, found only concurrent (and not cross-lagged) relations between teacher-student closeness and conflict and internalizing problems in adolescence (Pakarinen et al., 2018). Taken together, the longitudinal evidence suggests that externalizing problems can lead to poorer teacher relationships and that poorer teacher relationships are a risk factor for both externalizing and potentially also internalizing problems in adolescence. This points to teacher relationships as a potential mechanism through which internalizing-externalizing developmental cascades develop and progress throughout adolescence, in addition to peer problems.

In addition to the historically narrow focus on peer and academic problems as cascade mediators, a major issue in externalizing-internalizing cascade research concerns the potential mis-match between developmental cascade models and their statistical operationalization. Much of the strongest evidence for externalizing-internalizing cascade models to date comes from cross-lagged panel models fit to longitudinal data on internalizing, externalizing, and candidate mediators of their association. However, the parameters of the cross-lagged model represent aggregated between- and within- person effects, whereas developmental cascade models of externalizing and internalizing arguably refer to within-person processes (Curran et al., 2014). As such, accurate tests of externalizing-internalizing cascade hypotheses require statistical models that can separate within-person effects from between-person effects. The ALT-SR model described by Curran et al. (2014) provides a method of doing this. By fitting a cross-lagged structure to the residuals of a parallel process model (a latent growth curve model with multiple phenotypes with correlated intercepts), the ALT-SR partials out between-person variance, leaving cross-lagged parameters that better capture within-person processes. A similar disaggregation can also be achieved through a random-intercepts cross-lagged panel model (RI-CLPM; Hamaker et al., 2015). Only a handful of studies have, however, used a statistical design such as an ALT-SR or RI-CLPM that appropriately disaggregates between- and within-person effects and can, thus, provide unambiguous estimates of the developmental links between internalizing and externalizing problems (e.g., Oh et al., 2020). Furthermore, none of these studies examined mediators of the developmental relations between internalizing and externalizing problems.

Within externalizing problems, there is an important distinction between non-aggressive and aggressive problems, where the latter are considered indicative of more serious issues by the time of adolescence (Fairchild & Smaragdi, 2018). Further, aggression may be particularly liable to impact relationship qualities because of its direct, confrontive and interpersonal nature (as compared to other forms of externalizing problems involving rule-breaking). However, most previous developmental cascade studies have focused on externalizing problems more broadly, leaving the specific role of aggression as a risk for and potential outcome of internalizing problems an important outstanding question.

## Current Study

Previous developmental cascades research addressing internalizing and externalizing problem comorbidity has focused on peer and academic problems as mediators, yet evidence suggests that other significant relationships beyond peers, especially teachers may be an important additional mediator. Further, previous research has primarily relied on cross-lagged panel models which conflate the within-person effects that are the subject of developmental cascade theories with between-person effects. In the current study, it was, thus, hypothesized that internalizing and externalizing problems in early to middle adolescence would show reciprocal within-person relations when analyzed using longitudinal statistical models that disaggregrated within- and between-person relations. Further, it was hypothesized that these relations would be significantly, partially mediated by both peer and teacher relationships.

## Method

### Participants

Participants were drawn from the Zurich project on social development from childhood to adulthood (z-proso). Data are available from when participants were aged 7; however, the current analysis focusses on the adolescent period when the participants were aged 11, 13, and 15. Baseline (age 7) data collection occurred in 2004. The target sample of 1675 was defined based on a stratified random sampling procedure with school as the unit of sampling and school location and size as stratification variables. All children entering the first grade in the 56 schools (*n* = 1675) selected by the sampling procedure were invited to participate via their parents, with 1572 of those invited contributing data in at least one wave of the study. The current sample comprises 738 females and 785 males, or 97% of those who contributed at any wave and 91% of the target sample. Analyses of non-response described elsewhere (Eisner et al., 2019) suggest that initial participation and attrition was independent of a range of potentially relevant predictors, with the main exception being an increased likelihood of non-response among youth whose parents did not speak German (the official language of Zurich) as their first language. The study; however, found no relation between non-response and self-reported internalizing or aggression: the two main outcome variables in the current study. More information on z-proso, including recruitment, assessment procedures, previous publications, and sample descriptions can be found via the study’s website: http://www.jacobscenter.uzh.ch/en/research/zproso/aboutus.html.

### Ethical Considerations

Ethical approval was received from the Ethics Committee from the Faculty of Arts and Social Sciences of the University of Zurich. Active informed consent was provided by parents up until age 12, after which active informed consent was obtained from the participants directly. Parents could still choose to opt their child out until the age of 18.

### Measures

All items were self-reported and part of a broader questionnaire on psychosocial functioning administered in German, the official language of the study location, in paper and pencil format.

#### Internalizing problems

Internalizing problems were measured using the self-reported Social Behavior Questionnaire (SBQ; Tremblay et al., 1991). Based on the results of factor analytic investigations in a previous study (Murray et al., 2017), anxiety and depression items were combined into a single composite internalizing score. The composite comprised four items measuring anxiety (e.g., “I was worried”) and four items measuring depression (e.g., “I was sad without knowing why”). The reference period for the internalizing items was the previous month. Item responses were on a 5-point Likert scale from *never* to *very often*. Previous studies have examined the psychometric properties of the German version of the SBQ in the current sample. They have provided support for its reliability (Murray et al., 2019), factorial validity (Murray et al., 2019), and (at least metric) developmental invariance over adolescence (Murray et al., 2019) in the present sample. Internalizing composite scores were derived by averaging the eight item scores (Cronbach’s *α*_age 11_ = 0.79; *α*_age 13_ = 0.83; *α*_age 15_ = 0.83).

#### Aggression

Aggression was also measured using the SBQ. The aggression subscale comprised 12 items that covered multiple forms and functions of aggression: physical aggression (e.g., “You kicked, bit, or hit someone else”), reactive aggression (e.g., “You hit someone when they tried to take something from you”), indirect aggression (e.g., “When you were mad at someone you said bad things about him/her behind their back”), oppositional aggression (e.g., “You hit or kicked your parents when you were angry”) and proactive aggression (e.g., “You intimidated someone else to get what you wanted”). The measure, thus, reflects contemporary models of aggression by acknowledging both multiple forms of aggression, such as physical versus social aggression, and multiple functions of aggression, such as reactive versus proactive aggression (Marsee et al., 2011). The reference period for the aggression items was the previous six months for the first measurement wave (age 11) and for the previous 12 months for subsequent measurement waves. Composite scores for aggression were derived by averaging item scores across the 12 items (Cronbach’s *α*_age 11_ = 0.76; *α*_age 13_ = 0.84; *α*_age 15_ = 0.83).

#### Peer relationships

Peer relationships were measured as part of an assessment on school functioning. Peer relationships were measured using the same item format and using the sum of three items. These can be translated as “I get on well with the other kids in my class”, “we have a really good sense of community within the class”, and “the other kids in my class are nice to me”. Responses were recorded on a four-point Likert scale from *fully untrue* to *fully true*. The sum of responses to these three items was used in the statistical models (Cronbach’s *α*_age 11_ = 0.78; *α*_age 13_ = 0.78; *α*_age 15_ = 0 .79).

#### Teacher relationships

Current teacher relationships were measured as part of the same school functioning assessment as peer relationships. Participants were instructed to give an average assessment across all teachers if they had more than one teacher. They were measured using three items, which can be translated as “my teacher treats me fairly”, “my teacher helps me when necessary”, and “I get on well with my teacher”. Responses were recorded on a four-point Likert scale from *fully untrue* to *fully true*. The sum of these three items was used in the analysis (Cronbach’s *α*_age 11_ = 0.78; *α*_age 13_ = 0.77; *α*_age 15_ = 0.82).

### Statistical Procedure

An autoregressive latent trajectory model with structured residuals (ALT-SR; Curran et al., 2014) was used to examine the within-person cross-lagged relations between aggression, internalizing, teacher relationships and peer relationships. This model fits a cross-lagged structure to the residuals of a latent growth curve model with fixed slopes and random intercepts. The intercept factors are allowed to covary. Internalizing, aggression, peer relationships, and teacher relationships at all three time points (age 11, 13, and 15) were included in the model. In the latent growth curve part of the model, time intervals were fixed proportional to the distances between measurement waves. In the cross-lagged panel part of the model, all first-order autoregressive, first-order cross-lagged effects, and within time point (residual) covariances were included in the model.

Mediated within-person effects from aggression to internalizing and internalizing to aggression via both peer and teacher relationships were also included in the model. To test for longitudinal mediation, second-order cross-lagged effects were also included between internalizing at time 3 and aggression at time 1, as well as internalizing at time 1 and aggression at time 3. These paths capture the direct effects of aggression on internalizing and internalizing on aggression respectively. The indirect effects of aggression on internalizing problems were evaluated by the coefficients representing the product of regressing age 15 internalizing on the age 13 mediators (peer and teacher relationships) and regressing the age 13 mediators on age 11 aggression. The indirect effects of internalizing problems on aggression were tested in an analogous fashion. In all cases observed (summed or average) composite scores were used rather than latent constructs because combining the ALT-SR with a latent measurement model tends to lead to estimation difficulties in the current dataset, due to the overall complexity of such a model. The model was fit using robust maximum likelihood (MLR) estimation in Mplus (Muthén & Muthén, 2015).

To assess the statistical significance of the coefficients representing the indirect paths, their standard errors were calculated using the delta method. Bootstrapped confidence intervals are not available with MLR, therefore, as a sensitivity check, the model was also estimated using standard maximum likelihood estimation and statistical significance of the indirect effects assessed using bootstrapped 95% confidence intervals. Gender differences were adjusted for by regressing each intercept factor on gender.

In using MLR, missing data were dealt with using full information maximum likelihood (FIML) estimation. FIML gives unbiased parameter estimates provided data are missing at random (MAR). It is not generally possible to know whether data are MAR as opposed to not missing at random (NMAR) as this requires information on the missing values; however, it can be speculated that it is unlikely that there are serious biasing effects of any departures from MAR given that the proportion of missingness is not substantial (91% of the initial target sample and 97% of the recruited sample provided data in at least one wave used in the current study; the Ns for each construct at each wave are provided in Table 1). If there is NMAR, it is likely that, over and above the relation between observed values and missingness, those highest in internalizing and aggression or lowest in peer and teacher relationship quality would have the strongest chances of being absent from the sample. This is based on the idea that individuals with mental health and severe behavioral problems are more likely to be absent from community-ascertained studies (e.g., Graaf et al., 2000). Similarly, it can be speculated that poor teacher and peer relationships may reduce the likelihood of engagement with a study such as z-proso in which data collection occurs within the school context.

**Table 1:** Descriptive statistics

	*N*	Mean	SD	Min	Max	Range	Skew	Kurtosis	Alpha
Age 11 aggression	1144	1.54	0.44	1.00	4.00	3.00	1.52	3.23	0.76
Age 11 internalizing	1144	2.04	0.66	1.00	4.63	3.63	0.75	0.51	0.79
Age 11 teacher relationships	1134	10.42	1.77	3.00	12.00	9.00	−1.26	1.65	0.78
Age 11 peer relationships	1130	10.03	1.79	3.00	12.00	9.00	−0.82	0.45	0.78
Age 13 aggression	1365	1.75	0.59	1.00	4.89	3.89	1.36	2.20	0.84
Age 13 internalizing	1365	2.19	0.73	1.00	5.00	4.00	0.78	0.50	0.83
Age 13 teacher relationships	1341	9.47	1.96	3.00	12.00	9.00	−0.72	0.41	0.77
Age 13 peer relationships	1330	9.88	1.84	3.00	12.00	9.00	−0.82	0.67	0.78
Age 15 aggression	1446	1.69	0.56	1.00	4.56	3.56	1.52	2.82	0.83
Age 15 internalizing	1446	2.33	0.78	1.00	5.00	4.00	0.58	−0.05	0.83
Age 15 teacher relationships	1438	9.20	2.00	3.00	12.00	9.00	−0.66	0.42	0.82
Age 15 peer relationships	1422	9.87	1.79	3.00	12.00	9.00	−0.74	0.49	0.79

Models were judged to fit well if TLI and CFI were >0.95, RMSEA was <0.05 (but acceptable if <0.08) (Hu & Bentler, 1999) and SRMR was <0.05 (Schermelleh-Engel et al., 2003).

## Results

### Descriptive Statistics

Descriptive statistics are provided in Table 1. The correlations between aggression, internalizing, and the candidate mediators across the three time points are provided in Table 2. Across the waves, internalizing and aggression correlations ranged from 0.08 to 0.26, showing a decrease over time.

**Table 2:** Observed variable Pearson correlation matrix

	1.	2.	3.	4.	5.	6.	7.	8.	9.	10.	11.	12.
1. Age 11 aggression	–											
2. Age 11 internalizing	0.26***	–										
3. Age 11 teacher relationship	−0.32***	−0.15***	–									
4. Age 11 peer relationships	−0.24***	−0.22***	0.36***	–								
5. Age 13 aggression	0.43***	0.06*	−0.22***	−0.12***	–							
6. Age 13 internalizing	0.07*	0.39***	−0.11***	−0.20***	0.16***	–						
7. Age 13 teacher relationship	−0.22***	−0.01	0.25***	0.14***	−0.32***	−0.16***	–					
8. Age 13 peer relationships	−0.15*	−0.04	0.11***	0.22***	−0.22***	−0.25***	0.34***	–				
9. Age 15 aggression	0.34***	<0.001	−0.12***	−0.01*	0.55***	0.06*	−0.18***	−0.10***	–			
10. Age 15 internalizing	0.01	0.33***	−0.06	−0.09***	0.03	0.55***	−0.15***	−0.12***	0.08***	–		
11. Age 15 teacher relationship	−0.14***	−0.02	0.14***	0.05	−0.17***	−0.08*	0.32***	0.13***	−0.23***	−0.13***	–	
12. Age 15 peer relationships	−0.07*	−0.02	0.09***	0.12***	−0.09***	−0.14***	0.15***	0.29***	−0.11***	−0.22***	0.30***	–

*p < 0.05; **p < 0.01; ***p < 0.001

### Developmental Cascade Model

**Fig. 1:**
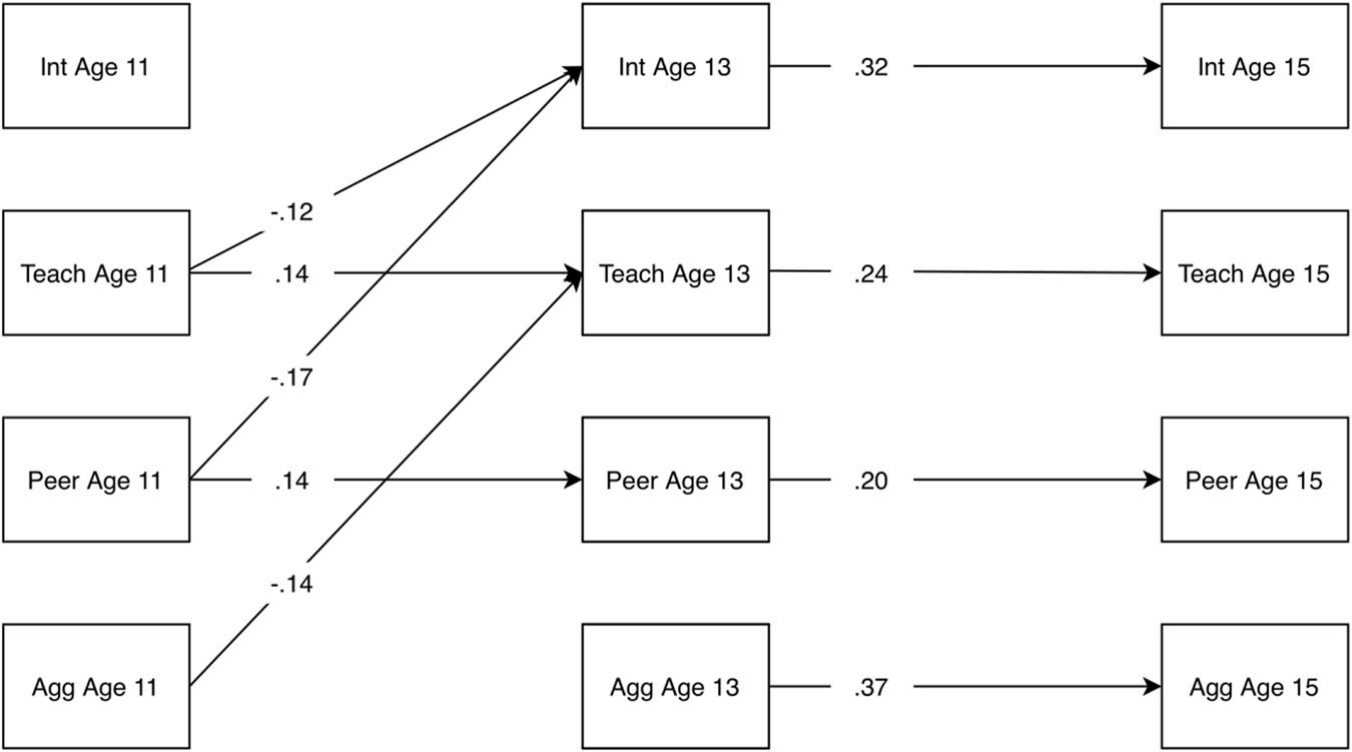
Autoregressive and cross-lagged parameters from ALT-SR model. Int internalizing, Teach teacher relationships, Peer peer relationships, Agg aggression. Statistically significant paths shown only. In addition, the latent growth curve and (residual) covariance parameters of the model are omitted for clarity. The latter are provided in Table 4

The ALT-SR showed poor fit according to RMSEA (0.09) and TLI (0.63) but good fit according to CFI (0.92) and SRMR (0.04). The poor fit according to TLI and RMSEA likely reflects a lack of model parsimony; however, non-significant paths were not trimmed because all were considered necessary to ensure a complete operationalization of the developmental cascade hypothesis being tested. Standardized autoregressive and cross-lagged parameters of the ALT-SR are provided in Table 3 and summarized in Fig. 1. Figure 1 includes only the statistically significant autoregressive and cross-lagged parameters, omitting all other parameters for clarity.

**Table 3:** Standardized autoregressive and cross-lagged parameter estimates from the ALT-SR model

Parameter	Estimate	SE	*p*
Age 13 aggression on age 11 aggression	0.119	0.081	0.142
Age 13 aggression on age 11 internalizing	0.048	0.125	0.698
Age 13 aggression on age 11 teacher relationships	−0.096	0.052	0.063
Age 13 aggression on age 11 peer relationships	−0.005	0.042	0.909
Age 13 internalizing on age 11 aggression	0.040	0.169	0.814
Age 13 internalizing on age 11 internalizing	0.095	0.076	0.208
Age 13 internalizing on age 11 teacher relationships	−0.119	0.055	0.029*
Age 13 internalizing on age 11 peer relationships	−0.174	0.052	0.001**
Age 13 peer relationships on age 11 aggression	−0.087	0.066	0.183
Age 13 peer relationships on age 11 internalizing	−0.012	0.053	0.825
Age 13 peer relationships on age 11 teacher relationships	−0.004	0.048	0.941
Age 13 peer relationships on age 11 peer relationships	0.142	0.054	0.008**
Age 13 teacher relationships on age 11 aggression	−0.141	0.067	0.035*
Age 13 teacher relationships on age 11 internalizing	0.056	0.056	0.319
Age 13 teacher relationships on age 11 teacher relationships	0.137	0.060	0.023*
Age 13 teacher relationships on age 11 peer relationships	0.039	0.049	0.432
Age 15 aggression on age 13 aggression	0.367	0.048	<0.001***
Age 15 aggression on age 13 internalizing	0.013	0.091	0.885
Age 15 aggression on age 13 teacher relationships	0.001	0.039	0.972
Age 15 aggression on age 13 peer relationships	−0.045	0.040	0.263
Age 15 aggression on age 11 internalizing	−0.064	0.087	0.457
Age 15 internalizing on age 13 aggression	0.004	0.076	0.961
Age 15 internalizing on age 13 internalizing	0.319	0.058	<0.001***
Age 15 internalizing on age 13 teacher relationships	−0.082	0.046	0.073
Age 15 internalizing on age 13 peer relationships	−0.037	0.039	0.347
Age 15 internalizing on age 11 aggression	0.026	0.114	0.822
Age 15 peer relationships on age 13 aggression	0.000	0.039	0.998
Age 15 peer relationships on age 13 internalizing	−0.091	0.048	0.058
Age 15 peer relationships on age 13 teacher relationships	0.041	0.042	0.333
Age 15 peer relationships on age 13 peer relationships	0.199	0.049	<0.001***
Age 15 teacher relationships on age 13 aggression	−0.026	0.043	0.551
Age 15 teacher relationships on age 13 internalizing	−0.053	0.048	0.275
Age 15 teacher relationships on age 13 teacher relationships	0.238	0.048	<0.001***
Age 15 teacher relationships on age 13 peer relationships	0.003	0.042	0.943

*ALT‐SR* autoregressive latent trajectory model with structured residuals, *SE* standard error

*p < 0.05; **p < 0.01; ***p < 0.001

Peer and teacher relationships were both relatively stable across both lags; however, aggression and internalizing autoregressive paths were significant only between ages 13 and 15. Across the first lag, poorer teacher and peer relationships at age 11 were both associated with higher levels of internalizing at age 13; while higher levels of aggression at age 11 were associated with poorer teacher relationships at age 13. Based on standard errors computed using the delta method, there was no significant indirect effect of aggression on internalizing problems mediated either by peer relationships (*β* = 0.003, *p* = 0.41) or teacher relationships (*β* = 0.012, *p* = 0.11), nor was their combined mediating effect statistically significant (*β* = 0.015, *p* = 0.078). Bootstrapped confidence intervals also confirmed that there was no significant indirect effect via peer relationships (95% CI = −0.005–0.015), nor via teacher relationships (95% CI = −0.002–0.029). Their combined mediating effect was also not statistically significant (95% CI = −0.003–0.034). Effects of internalizing problems on aggression were also not significantly mediated by peer relationships (*β* = 0.001, *p* = 0.828, bootstrapped 95% CI = −0.006–0.008), teacher relationships (*β* = 0.000, *p* = 0.972, bootstrapped 95% CI = −0.008–0.007), nor by their combined effect (*β* = 0.001, *p* = 0.851, 95% bootstrapped 95% CI = −0.010–0.010). The (residual) correlations between the constructs at each wave are provided in Table 4. These indicate moderate to strong within-person concurrent associations between all constructs. In summary, the hypothesized cascades between internalizing and externalizing problems via peer and teacher relationships were not supported.

**Table 4:** Concurrent (residual) correlations from ALT-SR model

	Age 11				Age 13				Age 15			
	1.	2.	3.	4.	1.	2.	3.	4.	1.	2.	3.	4.
1. Aggression	–				–				–			
2. Internalizing problems	0.34	–			0.24*	–			0.15*	–		
3. Teacher relationships	−0.28***	−0.22***	–		−0.28***	−0.19***	–		−0.18*	−0.14*	–	
4. Peer relationships	−0.24***	−0.27***	0.34***	–	−0.18***	−0.29***	0.30***		−0.07	−0.20*	0.27*	–

Age 13 and 15 correlations are residual associations

*p < 0.05; **p < 0.01; ***p < 0.001

### Sensitivity and Additional Analyses

As noted above, the same pattern of results emerged irrespective of whether models were estimated with MLR or with standard ML using bootstrapped confidence intervals to assess the statistical significance of indirect effects. Additional exploratory analyses were also conducted in order to provide further insights into the findings and why they may have differed from some previous studies that have reported developmental relations between internalizing and externalizing problems. First, an ALT-SR with no mediators confirmed that there were no significant within-person relations between internalizing problems and aggression in a model without peer and teacher relationships: https://osf.io/b3pn6/. Second, an ALT-SR without adjusting for gender was fit: https://osf.io/zebxk/. In this model there were also no significant cross-lagged effects. Third, a standard cross-lagged panel model was fit: https://osf.io/k462e/. In this model there was a significant negative cross-lagged effect of aggression at age 13 on anxiety at age 15. While these findings highlights the importance of avoiding the conflation of within- and between-person effects, they remain inconsistent with previous findings, that have found positive developmental relations between internalizing and externalizing problems. As a final set of sensitivity analyses gender-stratified analyses are also reported: https://osf.io/qhm3t/ and https://osf.io/ajpbz/. Though it had been determined based on Monte Carlo power analyses that these stratified analyses would likely be under-powered, these are reported as they may nonetheless be helpful to pool with other findings in future meta-analytic investigations. In the model fit to the data for females none of the cross-lagged effects were significant, except a negative effect of peer problems at age 11 on internalizing problems at age 13. In the model fit to the data for males, the only significant cross-lagged effects were of teacher relationships at age 11 on internalizing problems at age 13.

## Discussion

Previous studies have identified developmental cascades between internalizing and externalizing problems; however, the mediating mechanisms of these cascades are not fully understood. This is because these studies have tended to focus primarily on peer relationships and academic problems as mediators. Previous studies have also mostly relied on cross-lagged panel models that cannot disaggregate within- and between-person relations and there is a need to replicate these cascades in models that can isolate within-person developmental relations from potential between-person confounding. The results of the study suggested that neither peer relationships nor teacher relationships were significant mediators of internalizing-externalizing developmental cascades in isolation or in combination. The results did, however, suggest evidence of protective effects of positive peer and teacher relationships in early adolescence (age 11) on later internalizing problems (age 13). Importantly, the use of an ALT-SR (Curran et al., 2014) to disaggregate within- and between-person effects, rather than the previously more commonly used CLPM, permits greater confidence that these protective effects are not due to the within-person-stable/between-person-varying confounds.

The lack of any evidence for developmental cascades between aggression and internalizing problems suggests no support for either the dual failure model (Capaldi, 1992) or the acting out (Carlson & Cantwell, 1980) model of externalizing-internalizing comorbidity in the current data. The current study is not the first to report null or mixed results in relation to these theoretical models. Blain-Arcaro and Vaillancourt (2017), for example, found evidence for the dual failure model but not the acting out model. Meanwhile, other studies have found evidence for an externalizing-to-internalizing cascade consistent with the dual failure model but limited or no support for peer and academic problems as mediators (e.g., Leadbeater & Hoglund, 2009). However, as most previous studies have not used models that disaggregate within- and between-person effects, further work using longitudinal models such as RI-CLPM and ALT-SR will be helpful for clarifying the extent of empirical support for the dual failure and acting out models (Murray et al., 2020).

The current study did, however, identify a novel protective effect of teacher relationships at age 11. This finding requires replication in future research but suggests that interventions to improve teacher-student relationships in early adolescence could help mitigate the risks for the emergence of internalizing problems in adolescence. Several previous studies (e.g., Driscoll & Pianta, 2010) evaluating interventions targeting teacher-student relationships have indicated promising results with respect to their positive impacts on child psychosocial outcomes. However, these studies have tended to focused on the impact of improving teacher-student relationship on externalizing problems in primary school children and the potential of teacher-student relationship interventions for the prevention of internalizing problems in adolescence has been little explored. It would also be beneficial to explore in future research which relationship aspects are most important in serving as protective factors for student mental health, for example, closeness, warmth, emotional support, or a lack of conflict (Pössel et al., 2013).

The fact that the protective effect of teacher relationships was limited to the age 11 to age 13 lag may have a number of explanations. As adolescents move out of early adolescence, it is thought that the relative influence of adult support figures declines, while the influence of peers (Steinberg & Monahan, 2007) and intimate partners (Anderson et al., 2015) increases, therefore, it is conceivable that it reflects a declining influence of teachers. However, the fact that the effect of peer relationships was also limited to the age 11 to age 13 lag suggests that the time-limitation of this protective effect is more reflective of a generalized critical period for relational (and perhaps broader) influences on internalizing problems. An attenuated influence of both teacher and peer relationships would also be consistent with the fact that the peak in onset of internalizing problems appears to be around 13-14 years of age (Kessler et al., 2005), suggesting that the preceding period could be especially important for the effects of risk and protective factors.

The effect of peer relationships at age 11 on internalizing problems at age 13 replicates a large body of previous work suggesting that peer problems such as bullying victimization and rejection are important risk factors for internalizing problems (e.g., Arseneault, 2018). However, the current study is among only a small number to confirm that this association holds when taking into account possible between-person confounds (specifically factors that may vary between people but tend to be relatively stable within people over time). The current study, thus, adds important evidence for the negative impact of peer problems in early adolescence and further underlines the potential value of school-based programs to prevent peer problems such as bullying (Gaffney et al., 2019).

It is important to highlight the limitations of the current study. First, the available measures of peer and teacher relationships were relatively brief. This meant that it was not possible to identify the specific aspects of peer problems (e.g., rejection) and teacher relationships (e.g., emotional support) that were the active ingredients in the protective effect of positive relationships on later internalizing problems. Second, the measurement waves were around two years apart, which may not correspond to the timescales over which the developmental cascades hypothesized play out. In particular, it may be that the interplay between aggression, internalizing problems, and peer and teacher relationships operates on a shorter timescale, resulting in an under-estimation of the importance of the pathways between these constructs in the current study. Indeed, almost all concurrent within-person covariances were significant in the ALT-SR. Future studies employing variable time measurement intervals could help address the question of the optimal time lag for capturing the interplay between aggression, significant relationships, and internalizing problems. The hypothesized timescale of effects is an aspect of developmental cascade models that is poorly specified, and further work should also focus on clarifying this. Third, the sample used in the current study was of a size such that precise gender-stratified estimates could not be obtained due to insufficient statistical power (based on Monte Carlo power analyses). Given that there are well established gender differences in internalizing problems and aggression, exploration of these relations in males and females separately will be important in future studies. Finally, the study sample was subject to some attrition and while previous analyses have suggested that drop-out was unrelated to previous levels of aggression and internalizing problems, it is not possible to be sure that it was “not missing at random” (NMAR) in missing data mechanism terms (Rubin, 1976). To the extent that the data were NMAR, estimates of model parameters will be biased in a difficult-to-predict direction.

Strengths of the present study include the use of a large, well-characterized community-ascertained longitudinal sample with relatively low levels of non-response and little non-random non-response (Eisner et al., 2019); the application of statistical models that can disaggregate between- and within-person effects (Curran et al., 2014); and the use of measures that have been shown to reliably measure a wide range of aggression and internalizing symptom levels (Murray et al., 2019). This latter strength is important for ensuring that associations are not attenuated due to limited reliable ranges of measurement.

## Conclusion

Previous research has identified developmental cascades between externalizing and internalizing problems; however, these require replication at the within-person level. Further, the mediating mechanisms of the cascades are yet to be fully characterized, with most previous studies focusing only on peer and academic problems as intermediaries. The current study used an ALT-SR to disaggregate between- and within-person relations and found no support for developmental cascades between aggression and internalizing problems. This calls into question the applicability of developmental cascade models such as the dual failure model and acting out model to adolescence. Better relationships with peers and teachers at age 11 were, however, protective against internalizing problems at age 13, suggesting that relationships in early adolescence are key influences on the emergence of anxiety and depression.

## References

[CR1] Anderson SF, Salk RH, Hyde JS (2015). Stress in romantic relationships and adolescent depressive symptoms: Influence of parental support. Journal of Family Psychology.

[CR2] Arseneault L (2018). Annual research review: The persistent and pervasive impact of being bullied in childhood and adolescence: Implications for policy and practice. Journal of Child Psychology and Psychiatry.

[CR3] Barbot, B., & Hunter, S. R. (2012). Developmental changes in adolescence and risks for delinquency. In E. L. Grigorenko (Ed.), *Handbook of juvenile forensic psychology and psychiatry* (pp. 11–34). Springer US. 10.1007/978-1-4614-0905-2_2

[CR4] Blain-Arcaro C, Vaillancourt T (2017). Longitudinal associations between depression and aggression in children and adolescents. Journal of Abnormal Child Psychology.

[CR5] Capaldi DM (1992). Co-occurrence of conduct problems and depressive symptoms in early adolescent boys: II. A 2-year follow-up at Grade 8. Development and Psychopathology.

[CR6] Carlson GA, Cantwell DP (1980). Unmasking masked depression in children and adolescents. The American Journal of Psychiatry..

[CR7] Curran PJ, Howard AL, Bainter SA, Lane ST, McGinley JS (2014). The separation of between-person and within-person components of individual change over time: A latent curve model with structured residuals. Journal of Consulting and Clinical Psychology.

[CR8] Driscoll KC, Pianta RC (2010). Banking time in head start: Early efficacy of an intervention designed to promote supportive teacher–child relationships. Early Education and Development.

[CR9] Eisner NL, Murray AL, Eisner M, Ribeaud D (2019). A practical guide to the analysis of non-response and attrition in longitudinal research using a real data example. International Journal of Behavioral Development.

[CR10] Fairchild, G., & Smaragdi, A. (2018). The neurobiology of offending behavior in adolescence. In Beech, A.R., Carter, A.J., Mann, R.E., Rotshtein, P. *The Wiley Blackwell handbook of forensic neuroscience* (pp. 421–453). John Wiley & Sons, Ltd. 10.1002/9781118650868.ch16

[CR11] Gaffney H, Ttofi MM, Farrington DP (2019). Evaluating the effectiveness of school-bullying prevention programs: An updated meta-analytical review. Aggression and Violent Behavior.

[CR12] Graaf R, de, Bijl RV, Smit F, Ravelli A, Vollebergh WA (2000). Psychiatric and sociodemographic predictors of attrition in a longitudinal study The Netherlands Mental Health Survey and Incidence Study (NEMESIS). American Journal of Epidemiology.

[CR13] Hamaker EL, Kuiper RM, Grasman RP (2015). A critique of the cross-lagged panel model. Psychological Methods.

[CR14] Hu L, Bentler PM (1999). Cutoff criteria for fit indexes in covariance structure analysis: Conventional criteria versus new alternatives. Structural Equation Modeling: A Multidisciplinary Journal.

[CR15] Kessler RC, Berglund P, Demler O, Jin R, Merikangas KR, Walters EE (2005). Lifetime prevalence and age-of-onset distributions of DSM-IV disorders in the National Comorbidity Survey Replication. Archives of General Psychiatry.

[CR16] Leadbeater BJ, Hoglund WL (2009). The effects of peer victimization and physical aggression on changes in internalizing from first to third grade. Child Development.

[CR17] Marsee MA, Barry CT, Childs KK, Frick PJ, Kimonis ER, Muñoz LC, Aucoin KJ, Fassnacht GM, Kunimatsu MM, Lau KS (2011). Assessing the forms and functions of aggression using self-report: Factor structure and invariance of the Peer Conflict Scale in youths. Psychological Assessment.

[CR18] Masten AS, Cicchetti D (2010). Developmental cascades. Development and Psychopathology.

[CR19] Murray AL, Eisner M, Obsuth I, Ribeaud D (2017). Situating violent ideations within the landscape of mental health: Associations between violent ideations and dimensions of mental health. Psychiatry Research.

[CR20] Murray AL, Eisner M, Ribeaud D (2016). The development of the general factor of psychopathology ‘p factor’ through childhood and adolescence. Journal of Abnormal Child Psychology.

[CR21] Murray AL, Eisner M, Ribeaud D (2019). Can the Social Behavior Questionnaire help meet the need for dimensional, transdiagnostic measures of childhood and adolescent psychopathology?. European Journal of Psychological Assessment.

[CR22] Murray AL, Eisner M, Ribeaud D (2020). Within-person analysis of developmental cascades between externalising and internalising problems. Journal of Child Psychology and Psychiatry.

[CR23] Murray AL, Obsuth I, Eisner M, Ribeaud D (2019). Evaluating longitudinal invariance in dimensions of mental health across adolescence: An analysis of the Social Behavior Questionnaire. Assessment.

[CR24] Muthén, L. K., & Muthén, B. (2015). Mplus. The Comprehensive Modelling Program for Applied Researchers: User’s Guide, 5

[CR25] Obsuth I, Murray AL, Malti T, Sulger P, Ribeaud D, Eisner M (2017). A non-bipartite propensity score analysis of the effects of teacher–student relationships on adolescent problem and prosocial behavior. Journal of Youth and Adolescence.

[CR26] Oh Y, Greenberg MT, Willoughby MT (2020). Examining longitudinal associations between externalizing and internalizing behavior problems at within-and between-child levels. Journal of Abnormal Child Psychology.

[CR27] Pakarinen E, Silinskas G, Hamre BK, Metsäpelto R-L, Lerkkanen M-K, Poikkeus A-M, Nurmi J-E (2018). Cross-lagged associations between problem behaviors and teacher-student relationships in early adolescence. The Journal of Early Adolescence.

[CR28] Pössel P, Rudasill KM, Sawyer MG, Spence SH, Bjerg AC (2013). Associations between teacher emotional support and depressive symptoms in Australian adolescents: A 5-year longitudinal study. Developmental Psychology.

[CR29] Rapee, R. M., Oar, E. L., Johnco, C. J., Forbes, M. K., Fardouly, J., Magson, N. R., & Richardson, C. E. (2019). Adolescent development and risk for the onset of social-emotional disorders: A review and conceptual model. *Behaviour Research and Therapy*, 103501 10.1016/j.brat.2019.103501 31733812

[CR30] Rubin DB (1976). Inference and missing data. Biometrika.

[CR31] Schermelleh-Engel K, Moosbrugger H, Müller H (2003). Evaluating the fit of structural equation models: Tests of significance and descriptive goodness-of-fit measures. Methods of Psychological Research Online.

[CR32] Steinberg L, Monahan KC (2007). Age differences in resistance to peer influence. Developmental Psychology.

[CR33] Theimann M (2016). School as a space of socialization and prevention. European Journal of Criminology.

[CR34] Tremblay RE, Loeber R, Gagnon C, Charlebois P, Larivee S, LeBlanc M (1991). Disruptive boys with stable and unstable high fighting behavior patterns during junior elementary school. Journal of Abnormal Child Psychology.

[CR35] van Lier PA, Vitaro F, Barker ED, Brendgen M, Tremblay RE, Boivin M (2012). Peer victimization, poor academic achievement, and the link between childhood externalizing and internalizing problems. Child Development.

[CR36] Wertz J, Zavos H, Matthews T, Harvey K, Hunt A, Pariante CM, Arseneault L (2015). Why some children with externalising problems develop internalising symptoms: Testing two pathways in a genetically sensitive cohort study. Journal of Child Psychology and Psychiatry.

[CR37] Yu R, Branje S, Meeus W, Koot HM, Van Lier P, Fazel S (2018). Victimization mediates the longitudinal association between depressive symptoms and violent behaviors in adolescence. Journal of Abnormal Child Psychology.

